# Human monocytes subjected to ischaemia/reperfusion inhibit angiogenesis and wound healing in vitro

**DOI:** 10.1111/cpr.12753

**Published:** 2020-01-19

**Authors:** Lars Hummitzsch, Martin Albrecht, Karina Zitta, Katharina Hess, Kerstin Parczany, René Rusch, Jochen Cremer, Markus Steinfath, Assad Haneya, Fred Faendrich, Rouven Berndt

**Affiliations:** ^1^ Department of Anesthesiology and Intensive Care Medicine University Hospital of Schleswig‐Holstein Kiel Germany; ^2^ Institute of Neuropathology University Hospital Muenster Muenster Germany; ^3^ Department of Cardiovascular Surgery University Hospital of Schleswig‐Holstein Kiel Germany; ^4^ Department of Applied Cell Therapy University Hospital of Schleswig‐Holstein Kiel Germany

**Keywords:** cytokines, ischaemia/reperfusion injury, monocytes, neoangiogenesis, remodelling, wound healing

## Abstract

**Objectives:**

The sequence of initial tissue ischaemia and consecutive blood flow restoration leads to ischaemia/reperfusion (I/R) injury, which is typically characterized by a specific inflammatory response. Migrating monocytes seem to mediate the immune response in ischaemic tissues and influence detrimental as well as regenerative effects during I/R injury.

**Materials and Methods:**

To clarify the role of classical monocytes in I/R injury, isolated human monocytes were subjected to I/R in vitro (3 hours ischaemia followed by 24 hours of reperfusion). Cellular resilience, monocyte differentiation, cytokine secretion, as well as influence on endothelial tube formation, migration and cell recovery were investigated.

**Results:**

We show that I/R supported an enhanced resilience of monocytes and induced intracellular phosphorylation of the prosurvival molecules Erk1/2 and Akt. FACS analysis showed no major alteration in monocyte subtype differentiation and surface marker expression under I/R. Further, our experiments revealed that I/R changes the cytokine secretion pattern, release of angiogenesis associated proteins and MMP‐9 activity in supernatants of monocytes exposed to I/R. Supernatants from monocytes subjected to I/R attenuated endothelial tube formation as indicator for angiogenesis as well as endothelial cell migration and recovery.

**Conclusion:**

In summary, monocytes showed no significant change in cellular integrity and monocyte subtype after I/R. Functionally, monocytes might have a rather detrimental influence during the initial phase of I/R, suppressing endothelial cell migration and neoangiogenesis.

## 1 INTRODUCTION

Ischaemic organ injury is one of the major causes of death and disability.^1^ Main events leading to ischaemic organ injury in the clinical routine are thrombotic or arteriosclerotic vascular occlusions and/or stenosis, shock, inflammation and major trauma.^2^ The imbalance of oxygen supply and demand causes a temporal loss of function and change of cellular morphology within the affected tissue and can result in irreversible cell death.^3^, ^4^ To prevent further organ damage, an early restoration of blood flow is essential.^5^ Paradoxically, reperfusion often amplifies the initial tissue damage, resulting in so called ischaemia/reperfusion (I/R) injury. I/R injury is characterized by the release of metabolic intermediates and reactive oxygen species, which provoke an inflammatory immune response.^2^, ^6^ The infiltration of leucocytes and the release of cytokines within the damaged tissue further promote a specific local microenvironment characterized by cell degradation, tissue remodelling and neoangiogenesis.^2^, ^3^, ^5^

Due to their involvement in human immune defense, inflammation and tissue regeneration, cytokine production, antigen processing and cell transformation, circulating monocytes play a crucial role in I/R injury.^7^, ^8^, ^9^, ^10^ Although, high cell plasticity as well as their capability to differentiate into various subtypes under certain conditions are well‐documented characteristics of monocytes, little is known about the precise role of monocytes in I/R injury, and thus, monocyte function in neoangiogenesis, tissue regeneration and inflammatory response.^7^, ^10^, ^11^, ^12^ It has been assumed that changing levels in oxygen supply may influence differentiation, maturation and function of circulating and migrating monocytes.^7^, ^10^ Moreover, it was shown that neoangiogenesis correlates positively with the number of monocytes/macrophages in various ischaemic injury models, including myocardial infarction and stroke. Whether adverse or protective influences of the monocyte/macrophage axis predominate in injured tissue has been controversially discussed in various studies.^7^, ^13^, ^14^ Former experimental studies have assumed that ischaemic injury is significantly reduced in the absence of monocytes/macrophages, while other reports described positive effects of monocyte transplantation for tissue repair in cardiovascular and neural ischaemic disease and suggested a high cell plasticity, pluripotency and resilience of monocytes with a high regenerative potential.^7^, ^9^ Especially the pluripotency of monocytes and their putative capability for further cell differentiation, cell‐cell transformation and neoangiogenesis has been discussed controversially.^7^, ^8^, ^9^, ^11^

It has also been hypothesized that the monocyte phenotype and thus release of cytokines, proangiogenic factors and enzymes change dynamically from a proteolytic and pro‐inflammatory M1 phenotype to an anti‐inflammatory and regenerative M2 phenotype during I/R injury.^15^ The balance between proteolytic and regenerative monocyte function throughout the temporospatial course of ischaemia and reperfusion could potentially play a crucial role in the determination of final tissue injury and subsequent tissue regeneration. Therefore, a thorough understanding of the cellular and molecular mechanisms of I/R injury and monocyte function is essential for the development of novel treatment strategies for I/R injury and related diseases.^15^

Although, most studies have investigated the influence of short, isolated hypoxic episodes on monocyte phenotype and function, the current in vitro study was designed to analyse the whole period of initial ischaemia and following reperfusion, representing I/R injury in vivo. Accordingly, we employed an in vitro monocyte cell culture model representing the two phases (ischaemia and reperfusion) of I/R injury to analyse the cellular and molecular effects of I/R on monocytes. Further, the effects of culture media from human monocytes that were subjected to I/R in vitro (conditioned culture media) were evaluated on endothelial tube formation as indicator for angiogenesis as well as endothelial scratch assays for endothelial cell migration and recovery.

## 2 MATERIALS AND METHODS

### 2.1 Ethics

The study was approved by the local ethics committee of the University Medical Center Schleswig‐Holstein, Kiel, Germany (protocol identification: D519/18 and D518/13). All procedures were in accordance with the Helsinki Declaration of 2000.

### 2.2 Experimental setting

Briefly, peripheral blood monocytes were obtained from leukapheresis products from the Department of Transfusion Medicine (University Hospital Schleswig‐Holstein, Kiel, Germany) and isolated by Ficoll‐Paque PLUS (GE Healthcare) density gradient centrifugation and selective adherence to cell culture surfaces according to established protocols.^16^

**Figure 1:**
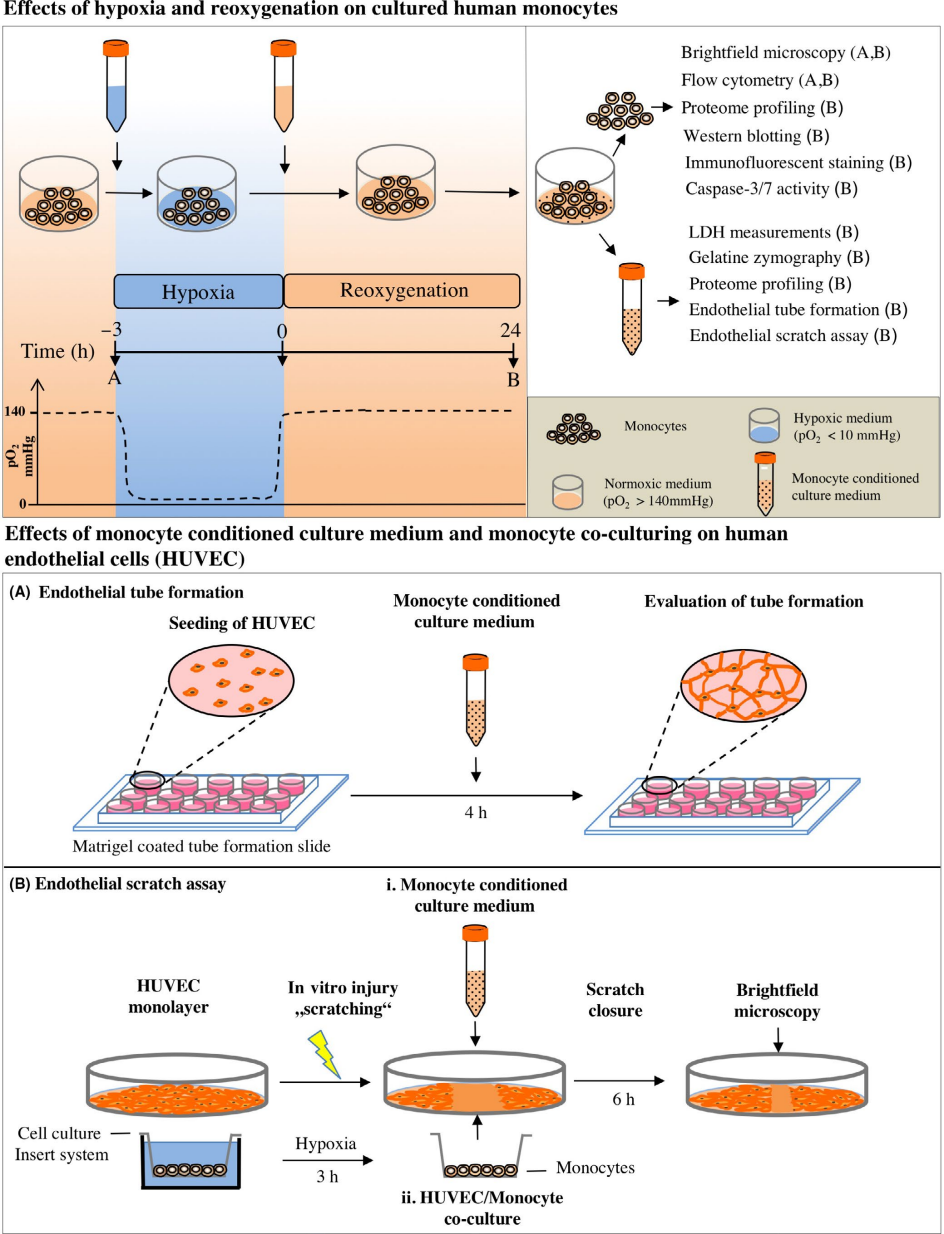
Schematic representation of the experimental setting

Monocytes (160 000 cells/cm^2^) were cultured in RPMI‐1640 medium containing 10% human AB‐serum, 2 mmol/L L‐glutamine, 100 U/mL penicillin and 100 μg/mL streptomycin. For all experiments, cells were allowed to adhere to the respective cell culture plates overnight. In vitro I/R was induced by using our previously described two‐enzyme system with minor modifications.^17^, ^18^ Briefly, for induction of ischaemia, cell culture medium was exchanged by medium containing 240 U/mL catalyse (Sigma‐Aldrich) and 4 U/mL glucose oxidase (Sigma‐Aldrich), resulting in a rapid decrease in partial pressure of oxygen (pO_2_) below 10 mm Hg. Moreover, using this system pH and glucose concentration in the culture medium decrease, resembling well the in vivo situation of I/R.^18^, ^19^ Hypoxic conditions were verified by using a tissue oxygen pressure monitor (LICOX CMP Oxygen Catheter; Integra). Pretests considering the increase in ischaemia‐inducible factors in monocytes and progression of cell damage during I/R were performed to evaluate the optimal duration of ischaemia (3 hours) and reperfusion (24 hours). Three major points in time were defined in the experiments: T(−3) represents the beginning of ischaemia, T(0) directly after ischaemia and T(24) the end of reperfusion (Figure 1). Ischaemia was terminated by replacing the hypoxic medium with standard culture medium, resulting in an immediate reoxygenation as well as increase in pH and glucose concentration in the cultures. Control experiments were performed under non‐ischaemic conditions by omitting the hypoxia inducing enzymes (glucose oxidase and catalase) from the respective culture media (Figure 1).

### 2.3 LDH cytotoxicity assay

The colorimetric Cytotoxicity Detection Kit PLUS (Roche) was used for the quantification of cell damage by measuring lactate dehydrogenase (LDH) activity released from cultured cells. Preparation of samples and measurements was performed according to the specifications provided by the manufacturer. Briefly, culture media were collected 24 hours after ischaemia and samples were stored at −20°C. The measurements were performed based on the protocol provided. For evaluation of total LDH, cells were lysed with 2% Triton X‐100 (Carl Roth) for 15 minutes at 37°C. LDH activity of the samples was analysed in 96‐well plates at 492 nm using an ELISA reader (Tecan) in combination with the magellan software v1.1. The measured activities in the samples were related to the activities of total LDH in the cultures.

### 2.4 Quantification of caspase‐3/7 activity

Apoptosis based on the activity of caspases‐3 and ‐7 was analysed using a rhodamine fluorometric assay (Apo‐One homogeneous caspase‐3/7 assay; Promega Corporation). 3.5 µg of total protein isolated from monocytes was used on the basis of the manufacturer's protocol. Fluorescent signals (Ex/Em 499/521 nm) were obtained with the fluorescence ELISA reader (Tecan) in combination with the software of magellan v1.1. Fluorescent arbitrary units (au) are depicted in the graphics.

### 2.5 Protein isolation and Western blotting

Protein isolation and Western blotting were performed as described earlier.^20^ Briefly, 28 μg of protein concentrate was mixed with 4× Laemmli buffer (8% SDS, 40% glycerol, 20% 2‐mercaptoethanol, 0.008% bromphenol blue, 0.25 mol/L Tris‐HCl, all from Sigma‐Aldrich) and incubated for 3 minutes at 95°C. Samples were separated by SDS‐PAGE and transferred to a PVDF membrane (Amersham Pharmacia Biotech). After 2 hours of blocking with TBST buffer containing 3% of bovine serum albumin (Carl Roth) at room temperature, the membranes were incubated overnight at 4°C with specific primary antibodies for Erk1/2 (1:10 000; Cell Signaling Technology), phospho‐Erk1/2 (1:8000; Cell Signaling Technology), Akt (1:2000; Cell Signaling Technology), phospho‐Akt (1:1000; Cell Signaling Technology), STAT5 (1:1000; R&D Systems) and phospho‐STAT5 (1:1000; R&D Systems). Signals from peroxidase‐conjugated secondary antibodies (anti‐rabbit; 1:20 000; Cell Signaling Technology) were detected using the ECL kit (ECL‐Plus Western blotting Detection Reagents; Amersham Pharmacia Biotech). Membranes were exposed to X‐ray‐films and intensities of the respective protein bands were analysed using the imagej software 1.41 (National Institutes of Health NIH).

### 2.6 Flow cytometry

To analyse putative functional and phenotype differentiation under I/R, monocytes from three donors were characterized by flow cytometry using the BD FACSCalibur™ cytometer (BD Biosciences). Specific antibodies and their corresponding isotypes (all from BD Bioscience) were directly conjugated with phycoerythrin (PE) for CD16 and its isotype mouse IgG1, with fluorescein isothiocyanate (FITC) for CD45 and its isotype mouse IgG1 and with allophycocyanin (APC) for CD14 and its isotype mouse IgG2a. The gating strategy consisted first to exclude the 7‐AAD stained cells (dead cells), scan (among the living cells) the presence of CD45+, subsequently identify the monocytes based on their size and granularity (on FSC/SSC profiles) and further discriminate the different monocyte populations (classic, inflammatory or patrolling monocytes) based on the proportion of CD14/CD16 markers on the gated cells. Monocytes with high Calcein‐AM (1 nmol/L; Thermo Fischer Scientific) fluorescent signal that excluded 7‐AAD stain (BD Bioscience) showed a >70% of viability with all treatments evaluated by FACS.

### 2.7 Immunofluorescence

Monocytes were seeded in 12‐well plates containing glass coverslips at a density of 3 × 10^5^ cells per well. After in vitro I/R treatments, attached cells were directly stained with calcein‐AM (2 µmol/L; Thermo Fischer Scientific) for living cells determination. Alternatively, cells were fixed, permeabilized and stained with rhodamine phalloidin and DAPI for structural determinations as follow. Coverslips with attached cells were washed with warm PBS, fixed with 4% PFA (Carl Roth GmbH) in PBS for 15 minutes at room temperature, permeabilized with 0.1% Triton‐X100 (Sigma‐Aldrich) for 15 minutes, washed in PBS and incubated with ImageIT FX signal enhancer (Invitrogen) for 30 minutes at room temperature, adding afterwards rhodamine phalloidin (165 nmol/L; Invitrogen) in PBS containing 1% albumin for 30 minutes in the dark. Coverslips were washed once more with PBS, inversely mounted on microscope slides with ProLongTM Diamond antifade mountant containing DAPI (Invitrogen). Slides were cured for 24 hours at room temperature before observation. Images were acquired using a Leica DM2000 LED fluorescent microscope equipped with DAPI, L5 and rhodamine filter cubes, a HC PL FLUOTAR 40×/0.80 objective, and a Leica DFC7000 T fluorescence camera, using the las Image Overlay software.

### 2.8 Proteome profiling arrays

Proteome profiling was performed using human angiogenesis arrays (ARY007; R&D Systems) and human XL cytokine arrays (ARY022B; R&D Systems) according to manufacturer's protocol provided with the assay kit. After culturing monocytes as described above, 200 μg of pooled samples of protein concentrate (intracellular proteins) and 500 µL (cytokine array) or 600 µL (angiogenesis array) of pooled cell culture medium (secreted proteins) was applied to the respective array membrane (angiogenesis array: N = 8; XL cytokine array: N = 13). Expression levels of 55 angiogenesis associated proteins and 160 cytokines were evaluated by densitometric analyses of the arrays using the imagej 1.41 software (NIH). For each spot on the membrane, the optical density was measured and the cut‐off signal level was set to 10% of the respective reference spots. Only regulations of more than 20% were considered as relevant and were further analysed.

### 2.9 Isolation of human umbilical vein endothelial cells (HUVEC)

Human umbilical vein endothelial cells were isolated from umbilical cords as described previously^21^ and cultured in endothelial cell growth medium ECGM (PromoCell) supplemented with 4 μL/mL of endothelial cell growth supplement, 0.1 ng/mL epidermal growth factor, 1 ng/mL basic fibroblast growth factor, 90 μg/mL heparin, 1 μg/mL hydrocortisone (all from PromoCell) and 10% foetal bovine serum (Thermo Fisher). The cells were maintained in a humidified atmosphere (5% carbon dioxide/95% air) at 37°C. For further experiments, cells were detached using a mild cell detachment solution (Accutase; Innovative Cell Technologies) and seeded in respective cell culture plates.

### 2.10 Endothelial tube formation assays

Human umbilical vein endothelial cells were harvested as described above and resuspended in the respective monocyte conditioned supernatants. Concerning the optimal time point of tube formation, several pilot experiments were performed employing different time points and different cell densities. Based on these studies and based on the manufacturer's recommendations (Ibidi GmbH), 10 000 HUVEC cells per well and 4 hours incubation time were chosen for all tube formation experiments. Therefore, 110 μL of cell suspension was seeded into each well (10 000 cells/well) of tube formation chambers (Ibidi) coated with Matrigel™. Photomicrographs of the cells were taken after 4 hours of cultivation in (a) cell culture media from monocyte that were subjected to I/R in vitro (I/R conditioned media, IRCM), (b) cell culture media from monocyte that were subjected to normoxia in vitro (normoxia conditioned media, NCM) and (c) non‐conditioned control monocyte medium (control medium, CM). Angiogenic parameters (number of meshes, nodes, segments and junctions) were evaluated from each picture using the angiogenesis analyzer of the imagej software 1.41 (NIH) as described previously.^22^

To characterize the potential influence of MMP‐9 in neoangiogenesis, further preliminary experiments were performed by additional administration of different amounts of recombinant MMP‐9 and in a second experiment MMP‐9 Inhibitor I for HUVEC tube formation. First, recombinant MMP‐9 (Calbiochem; Millipore) at 0, 0.1, 1 and 10 µg/mL dissolved in control culture media or pooled monocytes normoxic supernatants were added on HUVEC for tube formation to test the hypothesis that a significant amount of MMP‐9 could reduce the angiogenic parameters observed in HUVEC cultures. Second, MMP‐9 Inhibitor I (2‐(N‐Benzyl‐4‐methoxyphenylsulfonamido)‐5‐((diethylamino)methyl)‐N‐hydroxy‐3‐methylbenzamide; Merck KGaA) was added at concentrations of 0, 10, 50 and 100 nmol/L, prepared in pooled monocytes conditioned culture medium (IRCM) to test the hypothesis that MMP‐9 inhibition leads to significant more HUVEC tube formation.

### 2.11 Endothelial scratch assays

To assess the effect of cell culture media from monocytes that were subjected to I/R on endothelial cell migration and recovery of a HUVEC monolayer from defined injury, HUVEC were seeded onto fibronectin‐coated 6‐well plates (30 000 cells/cm^2^) and were grown to confluence. A mechanical scratch was produced by scrapping of the endothelial cells in a straight line with a p200 pipette to create a defined cell‐free space within the endothelial monolayer. The optimal time point for the evaluation of endothelial migration across the wound scratch (6 hours) was determined in the context of our previous work.^23^

Afterwards, endothelial cells were cultured in different cell culture media: (a) cell culture media from monocyte that were subjected to I/R in vitro (I/R conditioned media, IRCM), (b) cell culture media from monocyte that were subjected to normoxia in vitro (normoxia conditioned media, NCM), (c) control monocyte medium (control medium, CM). In an additional set‐up, HUVEC were co‐cultured with differently pretreated monocytes: (d) monocytes that were subjected to I/R in vitro (co‐culture I/R monocytes, CoIRM), (e) monocytes that were subjected to normoxia in vitro (co‐culture normoxia monocytes, CoNM). For monocyte co‐culture, monocytes were seeded (160 000 cells/cm^2^) in 6‐well plate inserts (Sarstedt) and exposed to 3 hours of ischaemic or normoxic conditions (control) in vitro. Inserts were then transferred into the culture dishes of scratched HUVEC. Photomicrographs of the scratched areas were taken directly and 6 hours after scratch induction. The percentage of closed scratch area was measured by comparing the scratch areas in the respective images using the imagej software 1.41 (NIH).

### 2.12 Gelatin zymography

Gelatin zymography was performed as previously described.^24^ Briefly, 10 µL of cell culture supernatants was loaded and separated on 7% SDS polyacrylamide gels (containing 1 mg/mL gelatin) under non‐reducing conditions. After electrophoresis, gels were first incubated in 2.5% Triton X‐100 for 30 minutes to remove SDS, followed by incubation in Tris‐HCl (50 mmol/L, pH 7.5) containing CaCl_2_ (5 mmol/L) and ZnCl_2_ (1 mmol/L) overnight at 37°C. After Coomassie blue staining, white bands of lysis indicated digestion of gelatin by matrix metalloproteinases (MMPs). Densitometric analysis was performed using the imagej 1.41 software (NIH).

### 2.13 Statistical analyses

All values are expressed as mean ± standard deviation (SD). Categorical variables are presented as frequency distributions (n) and percentages (%). Data were analysed with graphpad prism version 5.01 for Windows (graphpad Software) and tested for normality using the D'Agostino‐Pearson test or Kolmogorov‐Smirnov test. If normality was not present, data were transformed (arcsin of square root of *x*) before using parametric tests. Statistical comparisons of two groups were performed using student's *t* test or one‐sample *t* test. A *P* value < .05 was considered significant.

## 3 RESULTS

### 3.1 I/R in vitro increases resilience of monocytes and induces phosphorylation of prosurvival molecules without major changes in monocyte subtypes

**Figure 2:**
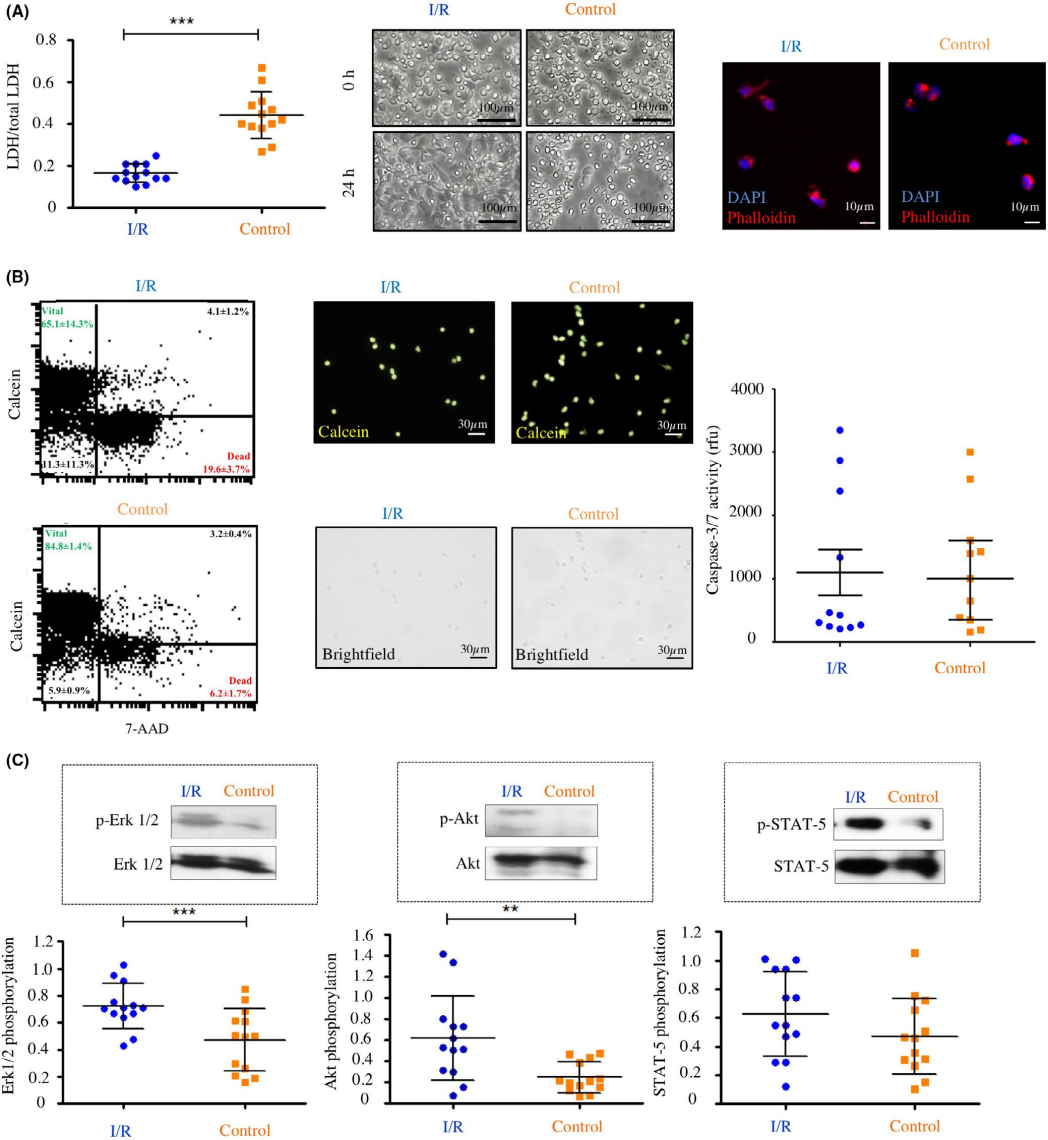
Effects of I/R on monocyte. A, Release of LDH into the cell culture medium (left), morphology of human monocytes in culture (middle) and staining for DNA (blue) and actin (red, right). B, Effects of I/R on monocyte vitality/apoptosis using flow cytometry (left, one representative experiment is shown, numbers in the scatter blots reflect N = 3 independent experiments), calcein vitality staining (middle) and Caspase‐3/7 activity (right). C, Assessment of the phosphorylation status of Erk1/2, Akt and STAT‐5 by Western blotting. I/R, Ischaemia/reperfusion; horizontal lines show the mean. Bars denote SD. **P < .01; ***P < .001

To quantify cellular disintegration of monocytes under I/R, LDH was measured in cell culture supernatants. I/R in vitro resulted in significantly lower LDH levels in cell culture supernatants after 24 hours compared to control conditions (I/R: 0.16 ± 0.04 au, control: 0.44 ± 0.11 au; *P* < .001, Figure 2A). Although, the morphology of the monocytes was slightly altered in the I/R group where cells represented a more spherical and attached phenotype (Figure 2A), immunofluorescent staining for actin did not reveal major differences between monocytes after ischaemia and under control conditions (Figure 2B). Additional flow cytometry experiments using live/dead staining (Calcein and 7‐AAD) as well as Caspase‐3/7 assays did not confirm the LDH measurements which suggested reduced cell damage after I/R (Figure 2B).

Phospho‐specific Western blotting experiments with protein lysates from monocytes after I/R revealed an increased phosphorylation of the intracellular kinases Erk‐1/2 (I/R: 0.72 ± 0.17 au, control: 0.47 ± 0.23 au; *P* < .001, Figure 2C) and Akt (I/R: 0.62 ± 0.40 au; normoxia: 0.25 ± 0.14 au; *P* < .01, Figure 2C). There was also a by trend increased phosphorylation status of the kinase STAT‐5 after I/R (I/R: 0.62 ± 0.30 au, control: 0.47 ± 0.27 au; *P* > .05, Figure 2C). Flow cytometric analysis of monocyte subtypes (classical monocytes: CD14+/16− vs non‐classical respectively intermediate monocytes: CD14+/CD16+) was performed after I/R and under control conditions. Under control conditions, both classical and non‐classical were represented in the investigated monocyte population (Appendix S1). After being subjected to I/R, there was only a minor shift towards the CD14+/CD16− monocyte subtype (Appendix S1).

### 3.2 I/R in vitro results in major changes of cytokine release and angiogenesis‐related protein expression

**Figure 3:**
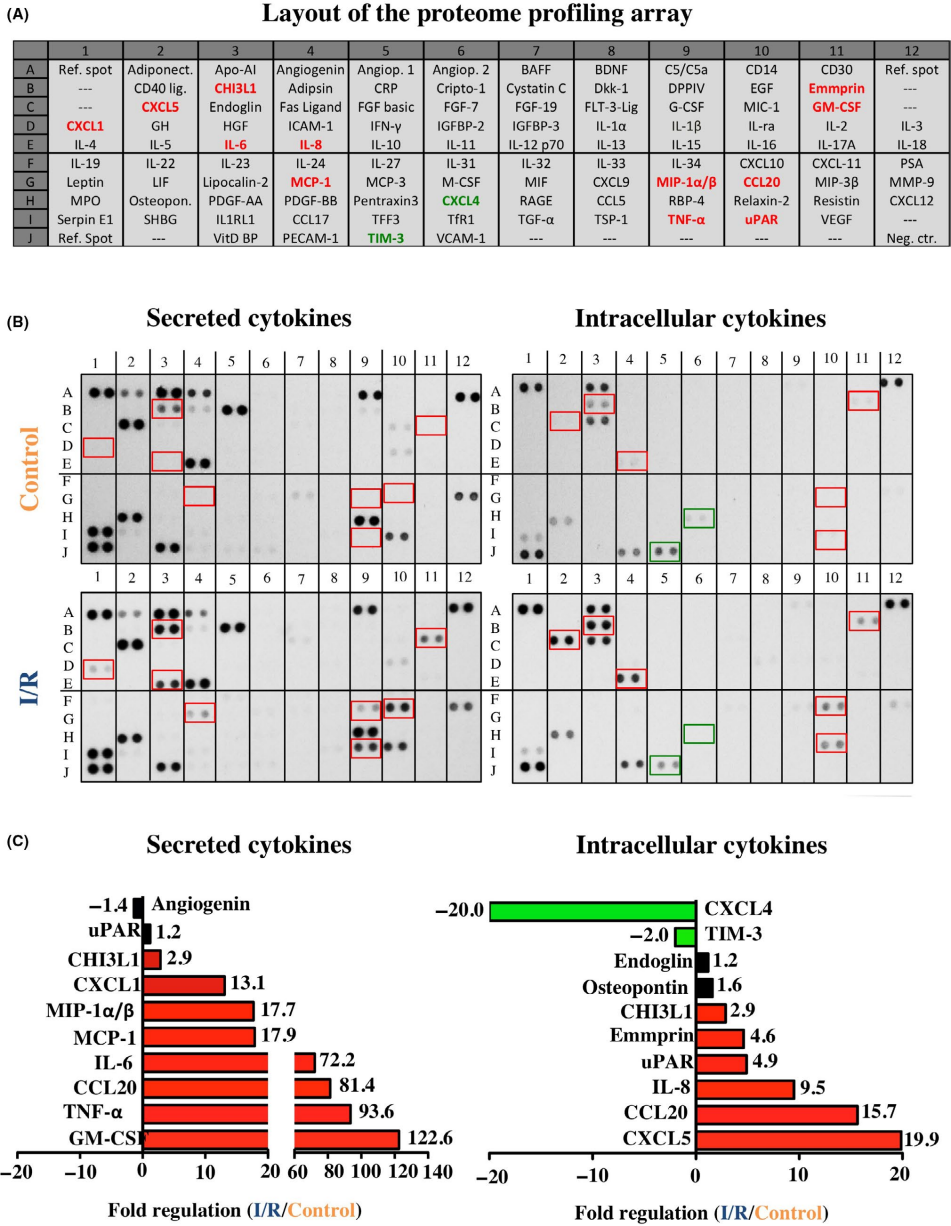
Profiling of secreted and intracellular cytokines. A, Analysed cytokines and location on the respective array membrane. B, Red font and rectangles represent intracellular and secreted cytokines that were more than two‐fold upregulated by I/R, while green font and rectangles indicate a more than two‐fold downregulation. C, Top ten most regulated proteins. All proteins are presented as duplicate spots on the respective array membrane. For a detailed description of the array proteins please refer to Appendix S2

Cytokine proteome profiler arrays were performed with total cell protein of monocytes (intracellular cytokines) as well as cell culture supernatants (secreted cytokines), as seen in Figure 3A,B. The respective results show that I/R in vitro lead to an up to 122‐fold upregulation and up to 20‐fold downregulation of various secreted and intracellular cytokines (Figure 3C). In detail, the most upregulated secreted cytokines were as follows: GM‐CSF (122‐fold), TNF‐α (93.6‐fold), CCL20 (81.4‐fold), Il‐6 (72.2‐fold), MCP‐1 (17.9‐fold), MIP‐1α/β (17.7‐fold), CXCL1 (13.1‐fold), CHI3L1 (2.9‐fold) and uPAR (1.2‐fold). In contrast, angiogenin was 1.4‐fold downregulated by I/R in vitro. The most upregulated intracellular cytokines were as follows: CXCL5 (19.9‐fold), CCl20 (15.7‐fold), IL‐8 (9.5‐fold), uPAR (4.9‐fold), EMMPRIN (4.6‐fold), CHI3L1 (2.9‐fold), Osteopontin (1.6‐fold), Endoglin (1.2‐fold). While intracellular expression of TIM‐3 (2‐fold) and CXCL4 (20‐fold) was downregulated by I/R in vitro (Figure 3B,C).

**Figure 4:**
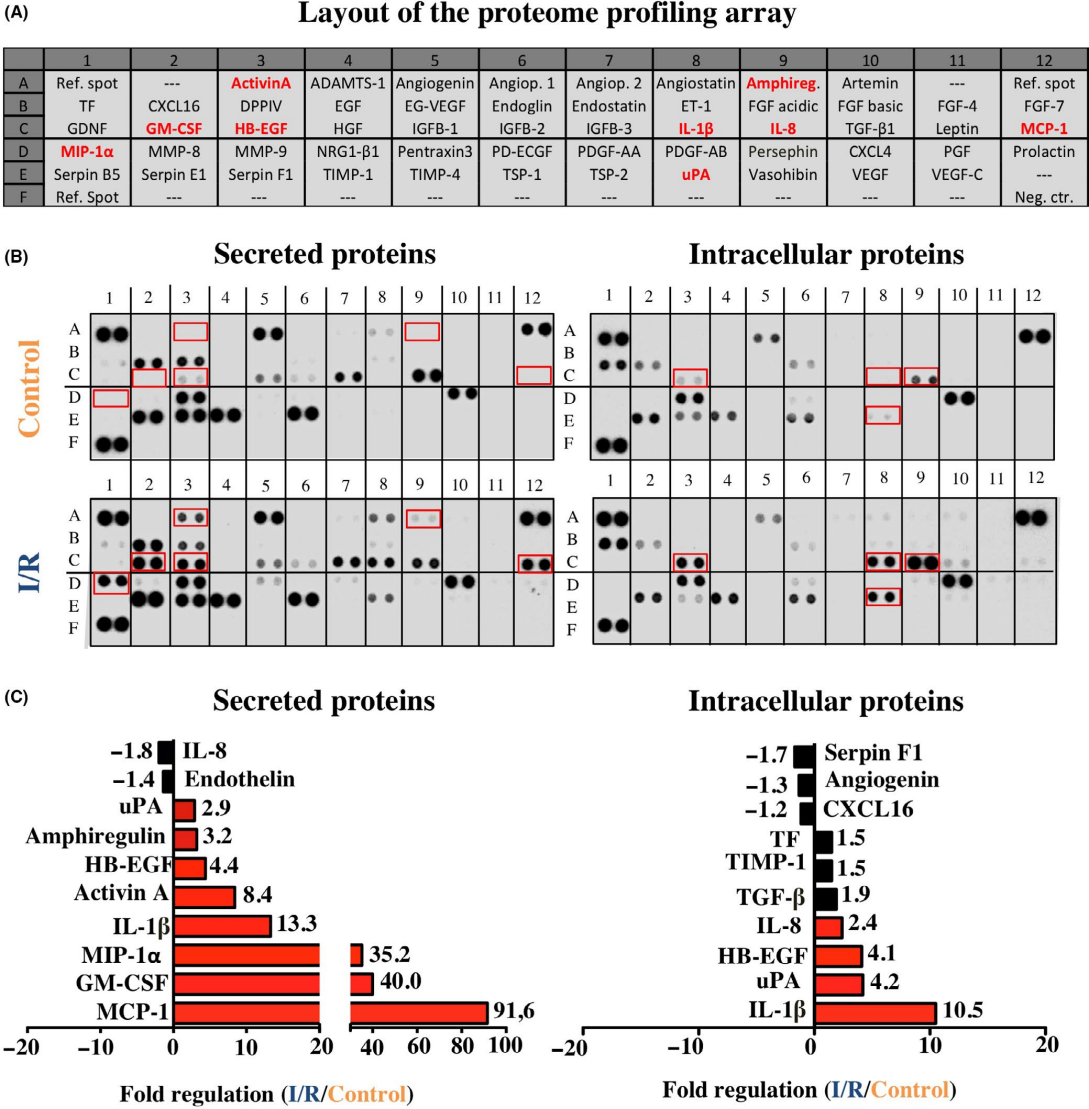
Profiling of secreted and intracellular angiogenesis‐related proteins. A, Analysed angiogenesis‐related proteins and location on the respective array membrane. B, Red font and rectangles represent intracellular and secreted cytokines that were more than two‐fold upregulated by I/R. C, Top ten most regulated proteins. All proteins are presented as duplicate spots on the respective array membrane. For a detailed description of the array proteins please refer to Appendix S3

In addition, angiogenesis proteome profiler arrays were performed with total cell protein of monocytes (intracellular factors) as well as cell culture supernatants (secreted factors), as seen in Figure 4A,B. The respective results show that I/R in vitro lead to an up to 91.6‐fold upregulation and up to 1.8‐fold downregulation of various angiogenic proteins (Figure 4C). In detail, the most upregulated secreted proteins were MCP‐1 (91.6‐fold), GM‐CSF (40‐fold), MIP‐1α (35.2‐fold), IL‐1β (13.5‐fold), Activin A (8.37‐fold), HB‐EGF (4.35‐fold), Amphiregulin (3.2‐fold), MMP‐8 (2.8‐fold), PTX‐3 (2.8‐fold) and Angiostatin (1.7‐fold). In contrast, IL‐8 (1.8‐fold) and Endothelin (1.4‐fold) were downregulated by I/R in vitro. The most upregulated intracellular proteins were IL‐1β (10.5‐fold), uPA (4.2‐fold) and Hb‐EGF (4.1‐fold). While intracellular expression of Serpin F1 (1.7‐fold), Angiogenin (1.3‐fold) and CXCL16 (1.2‐fold) was downregulated by I/R in vitro. For a detailed description of the array proteins please refer to Appendices S2 and S3.

### 3.3 Cell culture media derived from monocyte cultures subjected to I/R in vitro suppress angiogenesis and endothelial migration in vitro

**Figure 5:**
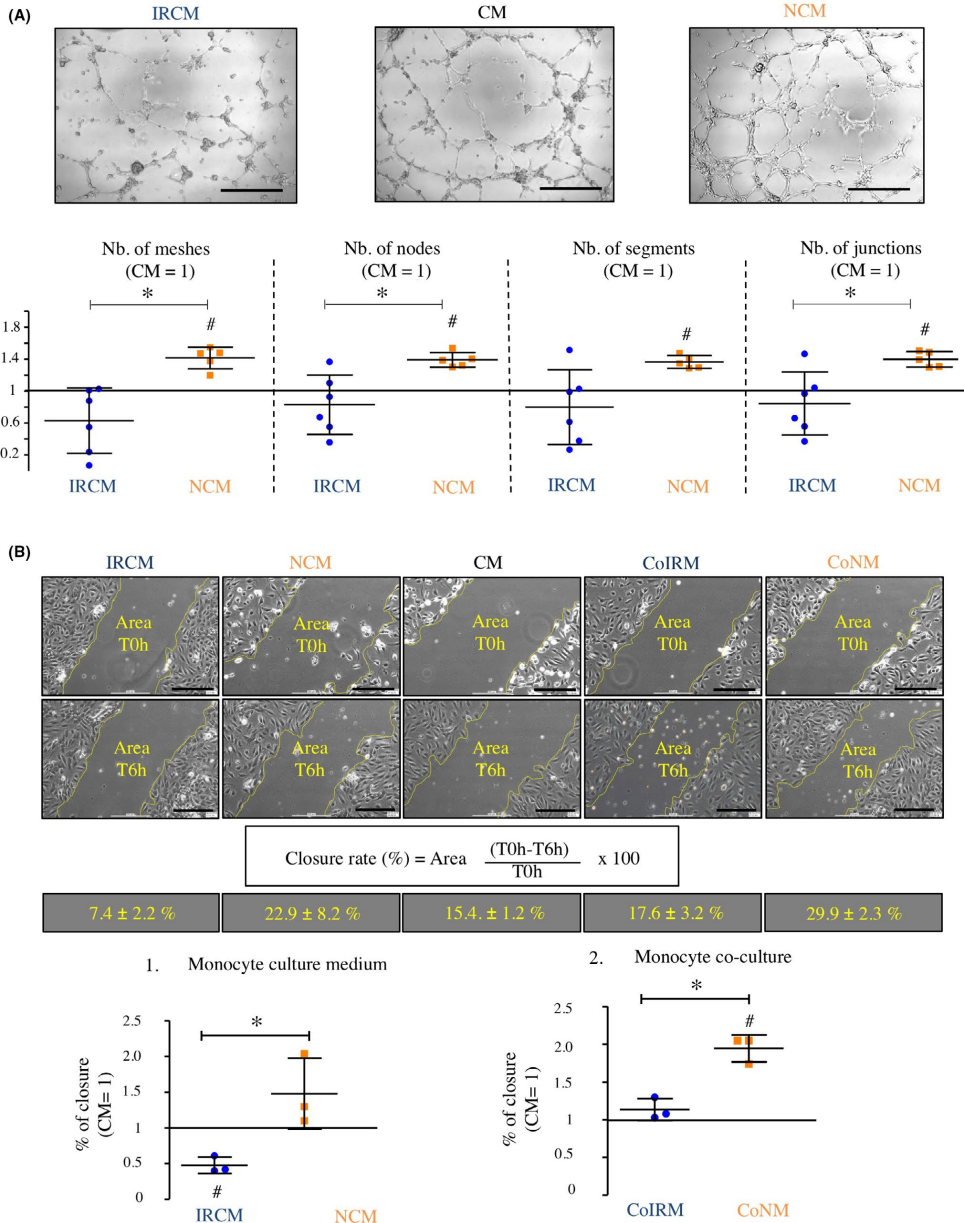
Effects of cell culture media derived from monocyte cultures subjected to I/R on endothelial tube formation and endothelial wound healing in vitro. A, Effects of cell culture media derived from monocyte cultures subjected to I/R on endothelial tube formation. B, Effects of cell culture media derived from monocyte cultures subjected to I/R and I/R conditioned monocytes on endothelial wound healing. Horizontal lines show the mean. Bars denote SD; *P < .05; ^#^ *P* < .05 (one‐sample *t* test vs CM). CM, control media; CoIRM, co‐culture I/R monocytes; CoNM, co‐culture normoxia monocytes; IRCM, ischaemia/reperfusion conditioned media; NCM, normoxia conditioned media

Tube formation assays for angiogenesis were performed with human umbilical vein cells (HUVEC) that were cultured on Matrigel‐coated dishes. Endothelial tube formation assays showed that cell culture media from monocytes that were subjected to I/R in vitro (I/R conditioned media, IRCM) induce less specific angiogenic parameters in HUVEC cultures compared to culture media from monocytes subjected to normoxic conditions (normoxia conditioned media, NCM): number of meshes, IRCM: 0.63 ± 0.41, NCM: 1.42 ± 0.14, *P* < .05; number of nodes, IRCM: 0.82 ± 0.37, NCM: 1.38 ± 0.09, *P* < .05; number of segments, IRCM: 0.80 ± 0.47, NCM: 1.37 ± 0.08, *P* > .05; number of junctions, IRCM: 0.84 ± 0.38, NCM: 1.38 ± 0.09, *P* < .05; Figure 5A).

To further evaluate the effects of cell culture media derived from monocyte cultures subjected to I/R in vitro on endothelial cell migration and recovery from defined injury, in vitro scratch assays were performed and the percentage of scratch closure was estimated 6 hours after the injury. IRCM showed significantly reduced closure rates of the scratched area compared to NCM (IRCM: 0.48 ± 0.12, NCM/CM: 1.48 ± 0.50, *P* < .05; Figure 5B). Co‐culture (insert system) of HUVEC with monocytes that were subjected to I/R (CoIRM) demonstrated less HUVEC migration into the scratched area compared to monocytes that were subjected to normoxia (CoNM) (CoIRM: 1.14 ± 0.14; CoNM/CM: 1.95 ± 0.18, *P* < .05; Figure 5B).

### 3.4 Secretome from monocytes subjected to I/R in vitro contain increased activity of MMP‐9

**Figure 6:**
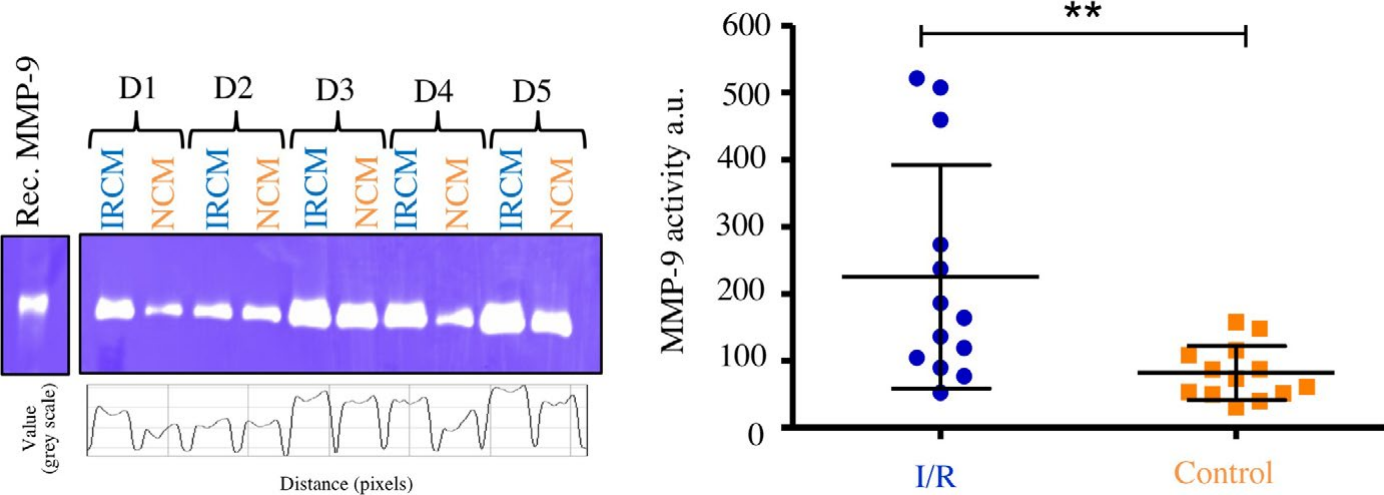
Effects of I/R on activity of secreted matrix metalloproteinases. Analyses of cell culture supernatants by gelatin zymography. Photos show representative experiments from five different donors (D1‐D5). Horizontal lines show the mean. Bars denote SD. **P < .01. I/R, ischaemia/reperfusion; IRCM, I/R conditioned media; NCM, normoxia conditioned media; rec. MMP‐9, recombinant matrix metalloproteinase‐9

Matrix metalloproteinases (MMPs) play a crucial role in remodelling, neoangiogenesis and wound healing.^25^ In order to further clarify the involvement of monocytes in remodelling and wound healing after I/R, gelatin zymography was performed to analyse the activity of potentially secreted MMPs. The results revealed that I/R conditioned media (IRCM) contained significantly more MMP‐9 activity when compared to normoxia conditioned media (NCM), suggesting that I/R leads to an increased secretion and/or activation of MMP‐9 by monocytes (IRCM, 224.93 ± 166.84 au; NCM, 81.60 ± 40.36 au; *P* < .01, Figure 6). To further clarify the potential role of MMP‐9 in neoangiogenesis, additional experiments were performed by adding different amounts of recombinant MMP‐9 to HUVEC cultures for tube formation and in a second experiment apply a MMP‐9 Inhibitor. Analyses of both experiments revealed no major changes between HUVEC tube formation under administration of MMP‐9 or inhibition of MMP‐9 activity (Appendix S4A,B).

## 4 DISCUSSION

Several authors have assumed a key role for the monocyte/macrophages‐axis in ischaemic microenvironments and during I/R injury by orchestrating human immune defense and inflammation, promoting tissue remodelling and neoangiogenesis.^26^, ^27^, ^28^, ^29^ However, it remains unclear if monocytes are mainly involved in the process of regeneration under I/R injury or merely mediate an immunological overreaction. Whereas monocytes under short‐term hypoxic conditions have been investigated before, monocyte phenotype and function during I/R injury have not been investigated in detail so far.^30^

Therefore, we employed an established in vitro I/R model representing the two phases (ischaemia and reperfusion) of I/R injury to analyse (a) monocyte viability and phosphorylation of key signalling/survival proteins, (b) monocyte differentiation and/or cell transformation and (c) secretion of cytokines/proteins involved in acute inflammatory response and neoangiogenesis. Moreover, the effects of culture media from monocytes that were subjected to I/R in vitro were evaluated in (d) HUVEC tube formation assays (in vitro angiogenesis) and in (e) endothelial scratch assays (endothelial migration and recovery). The current study only includes in vitro testing and might provide the basis for future animal and/or clinical studies.

To determine the viability of monocytes after I/R, apoptosis and cell damage were analysed. Interestingly, 3 hours of ischaemia followed by 24 hours of reperfusion did not influence apoptosis but resulted in significantly lower LDH levels in the culture supernatants compared to normoxia, suggesting an increased resilience of monocytes after I/R. Strong resistance of monocytes to I/R is essential to maintain specific cellular functions within the hypoxic microenvironment which is characterized by inflamed and damaged tissues enriched with cytotoxic inflammatory mediators and low levels of oxygen as well as vital substrates.^31^ In general, monocytes possess a limited life span and undergo spontaneous apoptosis during circulation.^32^ It is a well‐known fact that in the presence of specific pro‐inflammatory cytokines (TNF‐α, GM‐CSF) monocytes can interrupt their apoptotic programme and promote cellular survival by activation of the prosurvival kinase Akt.^32^, ^33^ Roiniotis et al demonstrated that monocytes exposed to 24 hours of hypoxia (without reperfusion) undergo a metabolic shift towards anaerobic glycolysis and show reduced apoptosis by activation of Akt.^30^ Accordingly, the positive effect of I/R on monocyte survival in our study was also associated with an increased phosphorylation of the prosurvival kinase Akt. In addition, we found an increased phosphorylation of Erk1/2, which has been suggested to also function as prosurvival kinase in various tissues and conditions.^34^, ^35^

The results of the necrosis and apoptosis assays did not fully correspond with the significantly lower LDH release by damaged monocytes after I/R. However, the verified level of necrosis was moderate and in case of Calcein/7‐AAD without statistical significance between the I/R and control groups. The results might point towards a moderate number of necrotic monocytes after I/R without significant involvement of apoptosis. This might be explained by the upregulation of the PI3K/Akt pathway, respectively, increased HIF1α expression via microRNA (ie *miR‐494*) inhibiting hypoxia‐induced apoptosis and consecutive downregulation of LDH release, as described before.^36^, ^37^

Contrary to former studies suggesting that altering oxygen supply could putatively influence monocyte subtype differentiation, our experiments revealed no major change of CD surface markers after I/R. Both classical and non‐classical monocyte subpopulation were equally represented in the experiments after I/R. Therefore, our data do not support the hypothesis that I/R could potentially influence detrimental or reparative capacity by monocyte subtype differentiation, as assumed before.^7^, ^10^, ^26^, ^28^

The initial pro‐inflammatory immune response during I/R injury is induced by various cytokines secreted by macrophages/monocytes and endothelial cells initiating the elimination of necrotic cells and matrix debris and maintaining the immunity barrier within the injured tissue.^38^ By contrast, the second phase of immunological reaction during I/R injury is determined by various cytokines and growth factors initiating tissue renewal and neoangiogenesis. However, exacerbation of the initial monocyte recruitment and cytokine release leads to further infiltration of leucocytes into the viable border zone of infarction and a subsequent increase in extracellular matrix production, inflammation, scar formation and finally organ dysfunction.^39^ The detailed molecular mechanisms of these opposing processes putatively mediated by migrating monocytes during I/R have not clarified so far. Hence, protein profiling of 105 different secreted cytokines and 55 angiogenesis associated proteins was investigated in the present experiments. It could be shown that various cytokines and angiogenesis‐related proteins were highly expressed and secreted under the influence of I/R in contrast to control conditions. Thus, monocytes seem to respond to I/R with the secretion of proteins involved in advanced inflammation (eg GM‐CSF, TNF‐α, CCL20, Il‐6), angiogenesis and tissue remodelling (eg MCP‐1, GM‐CSF, MIP‐1α and IL‐1β). It is commonly accepted that at least some effects of hypoxia are mediated by NF‐kB signalling and that Hypoxia Inducible Factor (HIF) and NF‐kB undergo extensive crosstalk.^40^, ^41^, ^42^ Current data of our proteome profiling arrays support this idea and suggest a possible involvement of NF‐kB signalling in monocytes, as several NF‐kB regulated target molecules such as IL‐8, MCP‐1, TNFα, GM‐CSF and IL1β are upregulated under hypoxic conditions.^43^, ^44^, ^45^

Especially, TNF‐α and Il‐6 mediated processes have been closely associated with ambivalent effects (detrimental vs beneficial) on tissue injury and tissue renewal in I/R injury. Accordingly**,** several authors described a protective effect for low levels of TNF‐α for the myocardium but also an increase in myocardial injury, contractile dysfunction, hypertrophy and fibrosis for high levels of TNFα after I/R injury.^43^, ^44^ Likewise, IL‐6 activity and its pro‐inflammatory pathways, depending on the cellular and temporal context, exert a wide range of diverse and competing effects including anti‐apoptotic, proliferative, growth‐inhibitory and differentiation‐inducing effects during I/R injury.^45^ Accordingly, the “Janus face” of inflammation after I/R injury is best exemplified by the presumption that TNF‐α and Il‐6 may contribute to tissue damage or protection depending on the temporospatial context of its expression.^45^ Our current results demonstrated that monocytes secret high levels of TNF‐α and IL‐6 during the first 24 hours of I/R pointing towards a putative predominantly detrimental role of monocytes in the initial phase of I/R injury**.** Therapeutic approaches targeting the inflammatory response of tissue injury are therefore promising treatment strategies attenuating the detrimental effects of I/R injury but further research on molecular mechanisms of the identified cytokines and the temporospatial context of their expression is necessary.^46^, ^47^, ^48^, ^49^, ^50^

Interestingly, proteome profiling arrays revealed that the release of several angiogenesis‐related proteins was altered in monocyte supernatants after I/R. Especially, MCP‐1, GM‐CSF, MIP‐1α, identified as some of the most enhanced proteins on both profiling arrays, have been described as potent effectors for remodelling and neoangiogenesis by promoting vascular endothelial growth factor‐A (VEGF‐A), endothelial progenitor cell (EPC) migration and recruitment of anti‐inflammatory macrophages (M2) at the site of ischaemic injury, where they participate in tissue recovery.^51^, ^52^, ^53^

Besides the mentioned proangiogenetic mediators, also a few cytokines with antiangiogenetic potential like Activin A, Angiostatin and Pentraxin‐3 was secreted into monocyte supernatants after I/R. The most upregulated antiangiogenic protein found in the culture media was Activin A, a multifunctional cytokine belonging to the TGF‐β family. While TGF‐β is a known initiator of angiogenesis, there is rising evidence, that Activin A exerts strong antiangiogenetic functions and can directly inhibit endothelial cell growth by interruption of cell cycle through downregulation of p21.^54^

To further investigate the influence of monocytes that were subjected I/R in vitro on angiogenesis and tissue recovery, endothelial tube formation and scratch assays with conditioned monocyte culture media were performed. Interestingly, the incubation of endothelial cells with cell culture medium from monocytes which were subjected to I/R in vitro (3 hours ischaemia, 24 hours reperfusion) resulted in reduced formation of capillary‐like structures and inhibited endothelial migration and scratch closure compared to supernatants from monocytes cultured under normoxic control conditions. Similar results were obtained in additional co‐culturing experiments, where endothelial cells were co‐cultured with monocytes that were previously exposed to 3 hours of ischaemia. In combination with our above mentioned proteome profiling results, these experiments suggest that under conditions of I/R, monocytes secret a characteristic inflammatory protein pattern which might be responsible for the observed attenuation of in vitro angiogenesis and endothelial cell migration and scratch closure.

We also show that culture media from monocytes that were subjected to I/R in vitro contain increased MMP‐9 activity. MMP‐9, a gelatinase that contributes to degradation of extracellular matrix, is highly expressed in disturbed wound healing and remodelling processes. However, inhibiting MMP‐9 activity or addition of recombinant MMP‐9 to HUVEC cultures did not affect angiogenic parameters. These ambivalent results might indicate that the detrimental effect of I/R conditioned media might not dependent solely on MMP‐9 activity or may involve complex pathways being not represented in this in vitro model. Further, the presence/absence of tissue inhibitor of metalloproteinase (TIMP) could also have influenced the results, as several authors have shown that MMP/TIMP ratio as well as the temporospatial context of MMP secretion and activity has both positive and negative effects on wound healing.^55^, ^56^, ^57^ Accordingly, further research, potentially in an animal model of wound healing/ tissue remodelling, is needed to clarify the ambiguous role of MMPs after I/R.

In summary, monocytes display a robust phenotype under conditions of I/R. Functionally, monocytes might have a rather detrimental influence during the initial phase of I/R, suppressing endothelial cell migration and neoangiogenesis. A more detailed understanding of the entire immunological response in the temporospatial context of I/R injury is necessary to unravel the underlying mechanisms responsible for the detrimental effects of monocytes infiltration and activation. A deeper understanding of these aspects could also further reveal new treatment options attenuating the adverse effects of initial ischaemia and subsequent reperfusion in various organs and diseases.

## CONFLICT OF INTEREST

The authors declare that they have no competing interests.

## AUTHOR CONTRIBUTIONS

LH, MA, FF, KZ and RB designed all experiments. All experiments were performed by LH, MA, RB, KP, KZ. All results of the experiments were analysed and evaluated by LH, KZ, KH, KP, RR, JC, MS, FF, AH, MA and RB. All authors interpreted results, prepared and contributed to the manuscript.

## Data Availability

All data are available on request due to privacy/ethical restrictions.
