# Supplementary figures and images for: Human monocytes subjected to ischaemia/reperfusion inhibit angiogenesis and wound healing in vitro
Source: Cell Prolif. 2020 Jan 19;53(2):e12753. doi: 10.1111/cpr.12753 (PMC7048205; doi:10.1111/cpr.12753)

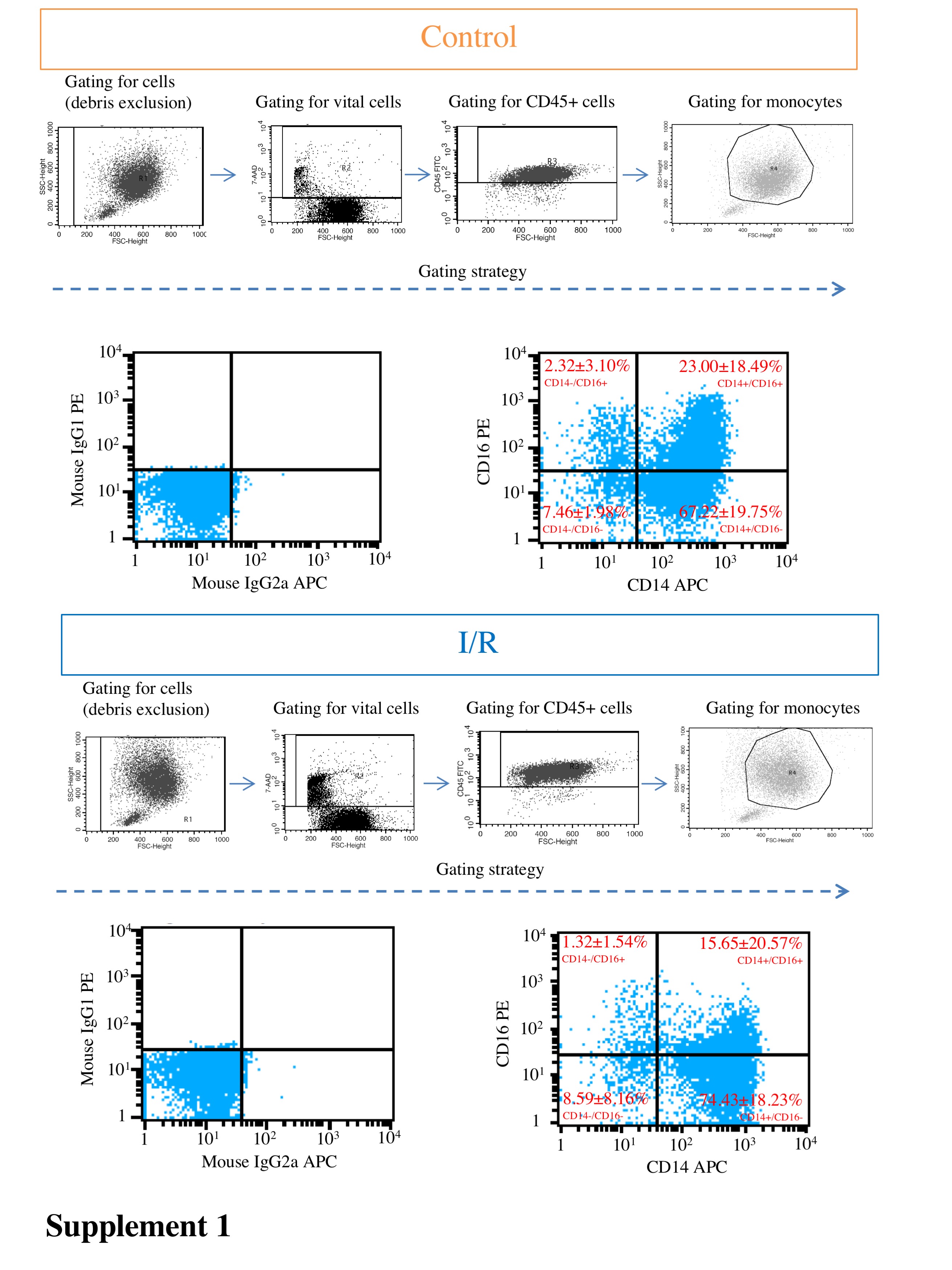

Supplement: Supplementary file 1 [file CPR-53-e12753-s001.jpeg]

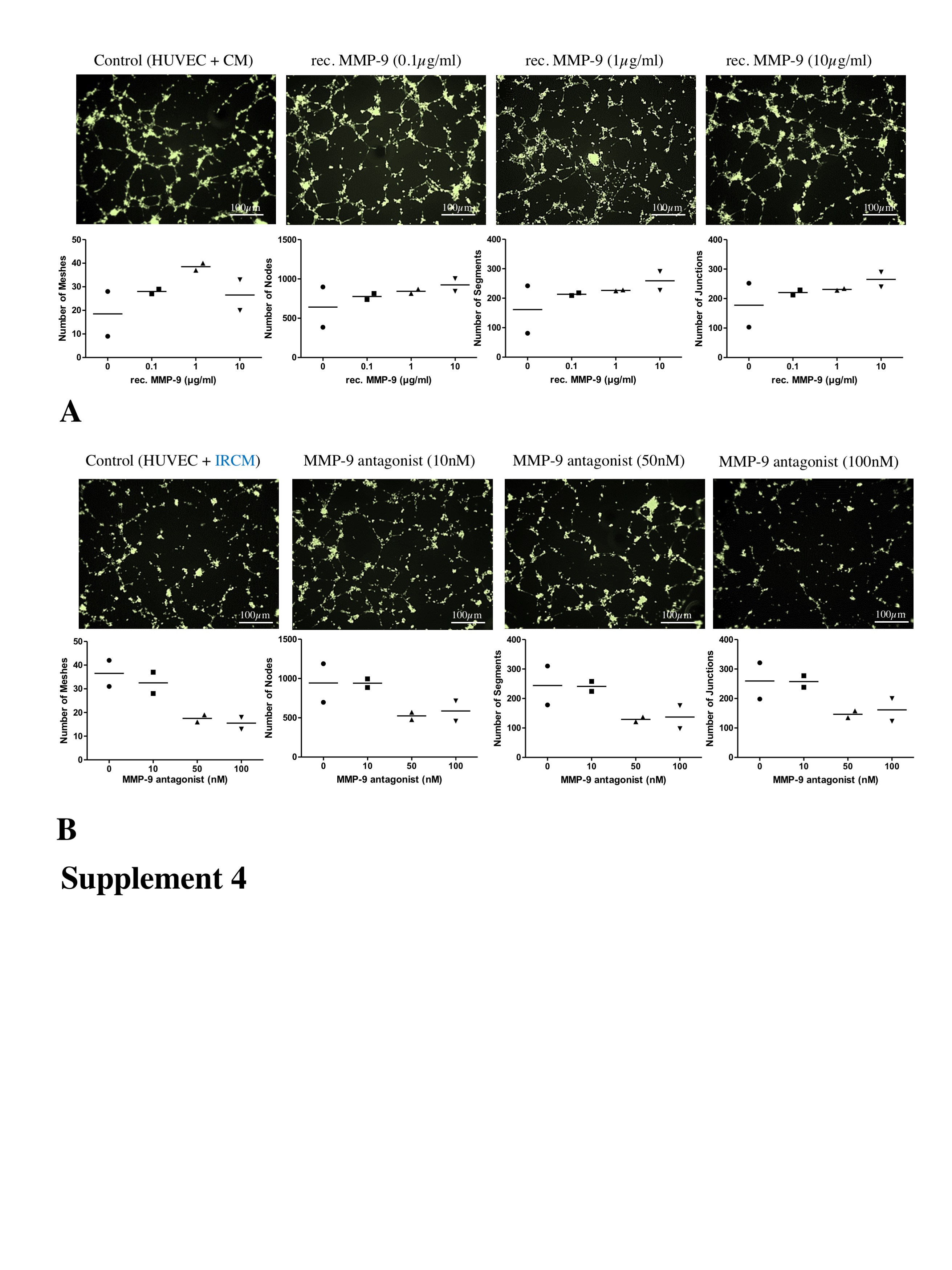

Supplement: Supplementary file 4 [file CPR-53-e12753-s004.jpeg]
