# Supplementary material for: Human monocytes subjected to ischaemia/reperfusion inhibit angiogenesis and wound healing in vitro
Source: Cell Prolif. 2020 Jan 19;53(2):e12753. doi: 10.1111/cpr.12753 (PMC7048205; doi:10.1111/cpr.12753)
Supplement: Supplementary file 2 [file CPR-53-e12753-s002.docx]

| **Coordinate** | **Abbreviation** | **Name (alternative nomenclature)** |
| --- | --- | --- |
| **A1; A12; J1** | Ref. spot | Reference spot |
| **A2** | Adiponect. | Adiponectin |
| **A3** | Apo-AI | Apolipoprotein A-I |
| **A4** | Angiogenin | Angiogenin |
| **A5** | Angiop. 1 | Angiopoetin 1 |
| **A6** | Angiop. 2 | Angiopoetin 2 |
| **A7** | BAFF | B-Cell activating factor |
| **A8** | BDNF | Brain-derived neurotrophic factor |
| **A9** | C5/C5a | Complement component C5/C5a |
| **A10** | CD14 | Cluster of differentiation 14 |
| **A11** | CD30 | Cluster of differentiation 30 (TNF-receptor superfamily member 8; TNFRSF8) |
| **B2** | CD40 lig. | CD40-ligand (TNF-receptor superfamily member 5; TNFSF5) |
| **B3** | CHI3L1 | Chitinase 3-like 1 |
| **B4** | Adipsin | Adipsin (Complement factor D; CFD) |
| **B5** | CRP | C-reaktive-protein |
| **B6** | Cripto-1 | Cripto-1 (Teratocarcinoma-derived Growth Factor; TDGF-1) |
| **B7** | Cystatin C | Cystatin C |
| **B8** | Dkk-1 | Dickkopf-1 |
| **B9** | DPPIV | Dipeptidyl-peptidase IV |
| **B10** | EGF | Epidermal growth factor |
| **B11** | Emmprin | Extracellular matrix metalloproteinase inducer (Basigin ; Cluster of differentiation 147; CD147) |
| **C2** | CXCL5 | C-X-C motif chemokine 5 |
| **C3** | Endoglin | Endoglin (Cluster of differentiation 105; CD105) |
| **C4** | Fas Ligand | Fas Ligand (TNF-receptor superfamily member 6; TNFSF6, Cluster of differentiation 178; CD178) |
| **C5** | FGF basic | Fibroblast growth factor basic (FGF-2) |
| **C6** | FGF-7 | Fibroblast growth factor-7 |
| **C7** | FGF-19 | Fibroblast growth factor-19 |
| **C8** | FLT-3-Lig | FMS-like tyrosine kinase 3 ligand |
| **C9** | G-CSF | Granulocyte-colony stimulating factor |
| **C10** | MIC-1 | Macrophage inhibitory cytokine-1 |
| **C11** | GM-CSF | Granulocyte-macrophage-colony stimulating factor |
| **D1** | CXCL-1 | C-X-C motif chemokine ligand 1 |
| **D2** | GH | Growth hormone (Somatotropin) |
| **D3** | HGF | Hepatocyte growth factor (Scatter factor) |
| **D4** | ICAM-1 | Intercellular adhesion molecule (Cluster of differentiation 54; CD54) |
| **D5** | IFN-γ | Interferon-γ |
| **D6** | IGFBP-2 | Insulin-like growth factor-binding protein 2 |
| **D7** | IGFBP-3 | Insulin-like growth factor-binding protein 3 |
| **D8** | IL-1α | Interleukin-1α |
| **D9** | IL-1β | Interleukin-1β |
| **D10** | IL-1ra | Interleukin-1 receptor antagonist |
| **D11** | IL-2 | Interleukin-2 |
| **D12** | IL-3 | Interleukin-3 |
| **E1** | IL-4 | Interleukin-4 |
| **E2** | IL-5 | Interleukin-5 |
| **E3** | IL-6 | Interleukin-6 |
| **E4** | IL-8 | Interleukin-8 (C-X-C motif chemokine ligand 8; CXCL8) |
| **E5** | IL-10 | Interleukin-10 |
| **E6** | IL-11 | Interleukin-11 |
| **E7** | IL-12 p70 | Interleukin-12 p70 |
| **E8** | IL-13 | Interleukin-13 |
| **E9** | IL-15 | Interleukin-15 |
| **E10** | IL-16 | Interleukin-16 |
| **E11** | IL-17A | Interleukin-17A |
| **E12** | IL-18 | Interleukin-18 |
| **F1** | IL-19 | Interleukin-19 |
| **F2** | IL-22 | Interleukin-22 |
| **F3** | IL-23 | Interleukin-23 |
| **F4** | IL-24 | Interleukin-24 |
| **F5** | IL-27 | Interleukin-27 |
| **F6** | IL-31 | Interleukin-31 |
| **F7** | IL-32 | Interleukin-32 |
| **F8** | IL-33 | Interleukin-33 |
| **F9** | IL-34 | Interleukin-34 |
| **F10** | CXCL10 | C-X-C motif chemokine ligand 10 (Interferon gamma-induced protein 10; IP-10) |
| **F11** | CXCL11 | C-X-C motif chemokine ligand 11 (Interferon-inducible T-cell alpha chemoattractant; I-TAC) |
| **F12** | PSA | Prostate-specific antigen (Kallikrein 3) |
| **G1** | Leptin | Leptin |
| **G2** | LIF | Leukemia inhibitory factor |
| **G3** | Lipocalin-2 | Lipocalin-2 (Neutrophil gelatinase-associated lipocalin; NGAL) |
| **G4** | MCP-1 | Monocyte chemoattractant protein 1(CC-chemokine ligand 2; CCL2) |
| **G5** | MCP-3 | Monocyte chemoattractant protein 3(CC-chemokine ligand 7; CCL7) |
| **G6** | M-CSF | Macrophage-colony stimulating factor |
| **G7** | MIF | Macrophage migration inhibitory factor |
| **G8** | CXCL9 | C-X-C motif chemokine ligand 9 (Monokine induced by gamma-interferon; MIG) |
| **G9** | MIP-1α/β | Macrophage inflammatory protein-1α (CC-chemokine ligand 3; CCL3), Macrophage inflammatory protein-1β (CC-chemokine ligand 4; CCL4), |
| **G10** | CCL20 | CC-chemokine ligand 20 (Macrophage inflammatory protein-3α; MIP-3α) |
| **G11** | MIP-3β | Macrophage inflammatory protein-3β (CC-chemokine ligand 19; CCL19) |
| **G12** | MMP-9 | Matrix metalloproteinase-9 |
| **H1** | MPO | Myeloperoxidase |
| **H2** | Osteopon. | Osteopontin |
| **H3** | PDGF-AA | Platelet-derived growth factor AA |
| **H4** | PDGF-BB | Platelet-derived growth factor BB |
| **H5** | Pentraxin3 | Pentraxin3 |
| **H6** | CXCL14 | C-X-C motif chemokine ligand 14 (Platelet factor 4; PF4) |
| **H7** | RAGE | Receptor for advanced glycation endproducts |
| **H8** | CCL5 | CC-chemokine ligand 5 (RANTES) |
| **H9** | RBP-4 | Retinol binding protein-4 |
| **H10** | Relaxin-2 | Relaxin-2 |
| **H11** | Resistin | Resistin (adipose tissue-specific secretory factor; ADSF) |
| **H12** | CXCL12 | C-X-C motif chemokine ligand 12 (stromal cell-derived factor 1; SDF-1) |
| **I1** | Serpin E1 | Serpin E1 (Plasminogen activator inhibitor-1 ; PAI-1) |
| **I2** | SHBG | Sex hormone-binding globulin |
| **I3** | IL1RL1 | Interleukin 1 receptor-like 1 |
| **I4** | CCL17 | CC-chemokine ligand 17 |
| **I5** | TFF3 | Trefoil factor 3 |
| **I6** | TfR1 | Transferrin receptor protein 1 |
| **I7** | TGF-α | Transforming growth factor α |
| **I8** | TSP-1 | Thrombospondin-1 |
| **I9** | TNF-α | Tumor necrosis factor α |
| **I10** | uPAR | Urokinase-type plasminogen activator receptor |
| **I11** | VEGF | Vascular endothelial growth factor |
| **J3** | VitD BP | Vitamin D binding protein |
| **J4** | PECAM-1 | Platelet endothelial cell adhesion molecule 1 (cluster of differentiation 31, CD31) |
| **J5** | TIM-3 | T-cell immunoglobulin and mucin-domain containing-3 |
| **J6** | VCAM-1 | Vascular cell adhesion molecule 1 (cluster of differentiation 106, CD106) |
| **J12** | Neg. ctr. | Negative controls |

**Supplement 2**
