# Supplementary material for: Human monocytes subjected to ischaemia/reperfusion inhibit angiogenesis and wound healing in vitro
Source: Cell Prolif. 2020 Jan 19;53(2):e12753. doi: 10.1111/cpr.12753 (PMC7048205; doi:10.1111/cpr.12753)
Supplement: Supplementary file 3 [file CPR-53-e12753-s003.docx]

| **Coordinate** | **Abbreviation** | **Name (alternative nomenclature)** |
| --- | --- | --- |
| **A1; A12; F1** | Ref. spot | Reference spot |
| **A3** | AktivinA | AktivinA |
| **A4** | ADAMTS1 | Angiogenin |
| **A5** | Angiogenin | Angiogenin |
| **A6** | Angiop. 1 | Angiopoetin 1 |
| **A7** | Angiop. 2 | Angiopoetin 2 |
| **A8** | Angiostatin | Angiostatin |
| **A9** | Amphireg. | Amphiregulin |
| **A10** | Artemin | Artemin |
| **B1** | TF | Tissue factor (Coagulation Factor III) |
| **B2** | CXCL-16 | C-X-C motif chemokine ligand 16 |
| **B3** | DPPIV | Dipeptidyl-peptidase IV |
| **B4** | EGF | Epidermal growth factor |
| **B5** | EG-VEGF | Endocrine gland derived vascular endothelial growth factor |
| **B6** | Endoglin | Endoglin (Cluster of differentiation 105; CD105) |
| **B7** | Endostatin | Endostatin |
| **B8** | ET-1 | Endothelin-1 |
| **B9** | FGF acidic | Fibroblast growth factor acidic |
| **B10** | FGF basic | Fibroblast growth factor basic (FGF-2) |
| **B11** | FGF-4 | Fibroblast growth factor-4 |
| **B12** | FGF-7 | Fibroblast growth factor-7 |
| **C1** | GDNF | Glial cell-derived neurotrophic factor |
| **C2** | GM-CSF | Granulocyte-macrophage-colony stimulating factor |
| C3 | HB-EGF | Heparin-binding epidermal growth factor |
| **C4** | HGF | Hepatocyte growth factor (Scatter factor) |
| **C5** | IGFBP-1 | Insulin-like growth factor-binding protein 1 |
| **C6** | IGFBP-2 | Insulin-like growth factor-binding protein 2 |
| **C7** | IGFBP-3 | Insulin-like growth factor-binding protein 3 |
| **C8** | IL-1β | Interleukin-1β |
| **C9** | IL-8 | Interleukin-8 (C-X-C motif chemokine ligand 8; CXCL8) |
| **C10** | TGF-β1 | Transforming growth factor-β1 |
| **C11** | Leptin | Leptin |
| **C12** | MCP-1 | Monocyte chemoattractant protein 1(CC-chemokine ligand 2; CCL2) |
| **D1** | MIP-1α | Macrophage inflammatory protein-1α (CC-chemokine ligand 3; CCL3) |
| **D2** | MMP-8 | Matrix metalloproteinase-8 |
| **D3** | MMP-9 | Matrix metalloproteinase-9 |
| **D4** | NRG1-β1 | Neuregulin1-β1 |
| **D5** | Pentraxin3 | Pentraxin3 |
| **D6** | PD-ECGF | Platelet-derived endothelial cell growth factor |
| **D7** | PDGF-AA | Platelet-derived growth factor AA |
| **D8** | PDGF-AB | Platelet-derived growth factor AB |
| **D9** | Persephin | Persephin |
| **D10** | CXCL4 | C-X-C motif chemokine ligand 4 |
| **D11** | PGF | Placental growth factor |
| **D12** | Prolactin | Prolactin |
| **E1** | Serpin B1 | Serpin B1 (Leukocyte elastase inhibitor; LEI) |
| **E2** | Serpin E1 | Serpin E1 (Plasminogen activator inhibitor-1 ; PAI-1) |
| **E3** | Serpin F1 | Serpin F1 (Pigment epithelium-derived factor; PEDF) |
| **E4** | TIMP-1 | Tissue inhibitor of metalloproteinases-1 |
| **E5** | TIMP-4 | Tissue inhibitor of metalloproteinases-4 |
| **E6** | TSP-1 | Thrombospondin-1 |
| **E7** | TSP-2 | Thrombospondin-2 |
| **E8** | uPA | Urokinase-type plasminogen activator |
| **E9** | Vasohibin | Vasohibin |
| **E10** | VEGF | Vascular endothelial growth factor |
| **E11** | VEGF-C | Vascular endothelial growth factor-C |
| **F12** | Neg. ctr. | Negative controls |

**Supplement 3**
